# Lessons Learned from a Nosocomial Outbreak of *Trichosporon asahii* in a High-Complexity University Hospital: Experience from Cali, Colombia †

**DOI:** 10.3390/jof12070506

**Published:** 2026-07-09

**Authors:** Jenny Patricia Muñoz-Lombo, Sandra Liliana Ossa, Gustavo Adolfo Clemen-Martínez, Raúl Andrés Vallejo-Serna

**Affiliations:** 1 Infectious Diseases Department, Hospital Universitario del Valle “Evaristo García”, Cali 760043, Colombia; 2 Infectious Diseases Department, Universidad del Valle, Cali 760043, Colombia; raulvallejomd@gmail.com; 3 Internal Medicine Department, Universidad del Valle, Cali 760043, Colombia; 4 Hospital Epidemiology Departament, Hospital Universitario del Valle “Evaristo García”, Cali 760043, Colombia; epidemiologia@correohuv.gov.co (S.L.O.); gustavo.clemen@correounivalle.edu.co (G.A.C.-M.)

**Keywords:** *Trichosporon asahii*, nosocomial outbreak, intensive care units, emerging mycoses, Colombia

## Abstract

**Background:** *Trichosporon asahii* is an emerging opportunistic yeast of growing concern in nosocomial settings, particularly in immunocompromised critically ill patients. Outbreaks in intensive care units remain infrequently reported, and environmental reservoirs are seldom fully characterized. **Methods:** A prospective outbreak investigation was conducted from 16 August to 29 October 2024 at a 496-bed high-complexity university hospital in Cali, Colombia. Case definitions distinguished healthcare-associated infection (HCAI) from colonization. Active surveillance included clinical cultures, environmental sampling of surfaces, biomedical equipment, and air conditioning duct systems. Microbiological identification was performed using MALDI-ToF mass spectrometry. **Results:** Nine cases were identified among 74 patients (6.76% attack rate); five were HCAIs, and four were colonizations. Overall mortality was 44%, though 0% was attributable to *T*. *asahii*. Primary risk factors included prolonged hospitalization, invasive devices, and broad-spectrum antibiotics. While environmental cultures were negative, maintenance records revealed unscheduled air duct cleaning and intermittent AC failures in the affected unit. **Conclusions:** Epidemiological evidence suggests that air conditioning malfunctions and temperature fluctuations facilitated fungal dispersal. The outbreak was contained through unit closure, hydrogen peroxide vaporization, and reinforced hand hygiene, highlighting the necessity of rigorous ventilation maintenance in high-complexity units.

## 1. Introduction

*Trichosporon asahii* is a ubiquitous basidiomycete yeast, widely distributed in soil, water, dairy products, and the skin and mucosal microbiota of humans. Its clinical relevance has increased considerably in the last decade, positioning it as the main agent of invasive trichosporonosis worldwide [1]. Similar to other opportunistic yeasts such as Candida auris and Candida parapsilosis, *T. asahii* possesses a remarkable ability to form biofilms on biotic and abiotic surfaces. This virulence factor facilitates the colonization of medical devices and compromises the efficacy of conventional antifungals, especially echinocandins [2].

Invasive *T*. *asahii* infections—including fungemia, fungal meningitis, peritonitis, and deep surgical site infections—have been described predominantly in patients with hematologic malignancies, diabetes mellitus, pulmonary disease, a history of transplantation, and a history of immunosuppressant use, among others [3]. However, there is growing evidence that critically ill patients without hematological pathology, especially those with prolonged stays in the ICU, constitute an underestimated risk group [4].

Nosocomial outbreaks of *Trichosporon* spp. are infrequently reported in the literature, but their consequences can be devastating. Previous studies have identified environmental reservoirs in dairy product distribution containers, catheters, and hospital ventilation systems as sources of clonal dissemination [5]. Molecular identification of the source through whole-genome sequencing or MLST (Multi-Locus Sequence Typing) is essential to confirm the epidemiological relationship between clinical and environmental strains [6].

Our institution is a high-complexity university hospital located in Santiago de Cali, Colombia, with a capacity of 492 beds and 74 adult intensive care unit (ICU) beds. It is the main referral center for southwestern Colombia, with an approximate annual admission of 244,000 patients.

In August 2024, the hospital epidemiology and infectious disease team identified an unusual cluster of *T*. *asahii* isolates in several units on the fourth floor of the hospital, prompting the activation of the outbreak investigation protocol. This article describes the epidemiological, microbiological, and environmental characteristics of this outbreak, with emphasis on the hypothesis of an environmental reservoir linked to the ventilation and air conditioning system.

## 2. Materials and Methods

A descriptive and prospective epidemiological study of a *T. asahii* outbreak was conducted. Cases were identified between 16 August and 25 October 2024, with a closing date of 5 November 2024 (one month without new infections). The final colonization case was documented on 1 October 2024. The investigation followed Ministry of Health protocols and was led by hospital epidemiology and infectious disease specialists. The study setting encompassed intensive care units (surgical, cardiovascular, and infectious diseases) and specialized hospital wards, with a total capacity of 74 beds.

Case Definition and surveillance: Three categories were established: Confirmed case (HAI), defined by the microbiological isolation of *T*. *asahii* from normally sterile sites with compatible clinical presentation according to CDC/NHSN criteria; Colonization case, identified through active screening of non-sterile samples; Exposed patients were defined as any patient hospitalized in the affected areas during the outbreak period. Prospective active surveillance was conducted through systematic weekly screening and retrospective analysis of the institutional microbiological database.

Microbiological identification: Species identification was performed using mass spectrometry (MALDI-ToF, Bruker Daltonics).

Environmental investigation and control: To ensure maximum accuracy, environmental and equipment sampling was performed by a specialized, certified microbiological analysis laboratory:

1. Air Surveillance: Active volumetric air sampling was conducted using a calibrated sieve-impactor sampler that aspirates air through a perforated head, impacting the viable particles directly onto standard agar plates. Post-collection, plates were incubated under standard conditions (25–30 °C for 5–7 days for fungi; 35–37 °C for 48 h for bacteria). Total colony-forming units (CFU) were quantified for both molds/yeasts and bacteria, utilizing an institutional safety benchmark of <30 CFU/30 min.
2. Surface Surveillance: High-touch surfaces and biomedical equipment were sampled using sterile rayon swabs with plastic shafts. Sampling was executed by firmly wiping a delimited area of 10 cm × 10 cm. Swabs were immediately immersed in sterile transport media and transported under controlled temperatures for inoculation onto Sabouraud Dextrose Agar and subsequent mycological incubation and identification.

Traceability was established for 4795 supplies, and ventilation systems, cleaning protocols, hand hygiene practices, and clinical care practices were evaluated. Control measures were implemented in stages after the outbreak was declared, and the outbreak was closed on November 5 after one month without new cases.

Statistical Analysis: Due to the descriptive nature and sample size of the outbreak cohort (*n* = 9), inferential statistical testing was not performed. Clinical and epidemiological characteristics were analyzed using descriptive statistics. Categorical variables are expressed as absolute frequencies and percentages. Continuous variables, including the length of hospital stay and duration of invasive device use prior to isolation, are expressed as medians and ranges. Critical outbreak indicators (such as attack rate, overall mortality rate, and pathogen-attributable case fatality rate) were calculated using standard epidemiological formulas.

## 3. Results

During the period from 16 August to 25 October 2024, nine patients were identified with positive cultures for *T. asahii* (Table 1). Of these, five presented with healthcare-associated infections (HAIs) and four with colonization detected through active surveillance (cases 4, 6, 7, and 8). A total of 74 patients were exposed.

The case descriptions are presented below:

**Case 1** (Infectious diseases ICU, 58 years old, male): Isolation of *T*. *asahii* in a blood culture taken on day 27 of hospitalization (16 August 2024), classified as a vascular line-associated bloodstream infection, with risk factors including the use of multiple devices (orotracheal intubation, central venous catheter), acute renal failure, and severe malnutrition. The patient passed away on 17 August, and the diagnosis was established post-mortem since the blood culture was reported positive on 20 August 2024.

**Case 2** (Surgical ICU, 32 years old, male): Simultaneous isolations in skin and soft tissue secretions taken on day 18 of hospitalization (21 August 2024), in the context of a postoperative intraperitoneal collection in a patient with penetrating abdominal trauma and multiple surgical interventions. Classified as an organ/space SSI. Following the isolation of *T. asahii*, antifungal therapy with amphotericin B was initiated on 29 August 2024; voriconazole (the first-line treatment) was initially avoided due to a history of substance abuse and hallucinations. However, due to subsequent amphotericin B unavailability, the patient was switched to voriconazole, completing a 2-week course with complete resolution of the collections. He was discharged alive after 63 days.

**Case 3** (Surgical ICU, 52 years old, female): Isolation in Cerebrospinal fluid sample taken on day 33 of hospitalization (26 August 2024), in the context of multiple external ventriculostomies due to Fisher IV subarachnoid hemorrhage. Classified as an organ/space SSI (fungal ventriculitis). On 29 August 2024, the external ventriculostomy was exchanged, and targeted therapy with intravenous liposomal amphotericin B (5 mg/kg/day; 350 mg/day) was initiated. However, the clinical course was severely complicated by the central nervous system involvement, manifesting as refractory status epilepticus with focal motor seizures (facial myoclonus) highly suggestive of Lance-Adams syndrome, which required multiple antiseizure medications. Subsequently, the patient developed persistent *Stenotrophomonas maltophilia* bacteremia. Given the irreversible clinical decline and poor prognosis, the case was evaluated by the Bioethics Committee, which recommended the redirection of therapeutic efforts. Death occurred 16 days after the diagnosis of *T. asahii* ventriculitis, after 71 days of hospitalization.

**Case 4** (Surgical ICU, 37 years old, male): isolation in culture from an abdominal collection taken on day 21 of hospitalization (5 September 2024), in the context of intra-abdominal complications secondary to a gunshot wound and multiple surgical interventions. Classified as an organ/space SSI. Targeted therapy was administered with intravenous liposomal amphotericin B (5 mg/kg/day; 350 mg/day) for 10 days due to the institutional unavailability of voriconazole. This treatment achieved an excellent clinical response and complete control of the abdominal infectious focus. Discharged alive after 44 days.

**Case 5** (Mixed ICU, 81 years old, male): history of supracondylar amputation of the right femur 7 years prior for benign pathology, isolation on day 15 in culture from the stump (25 October 2024) in the context of extensive electrical burn injury. Deep ISO classified. Antifungal therapy with posaconazole (300 mg BID on the first day, followed by 300 mg QD) was administered owing to the institutional unavailability of voriconazole. Serial surgical interventions were required for source and infection control. Although *T. asahii* persisted in early follow-up cultures, progressive clinical improvement was noted, and antifungal therapy was successfully discontinued after 28 days. Discharged alive after 124 days.

**Figure 1:**
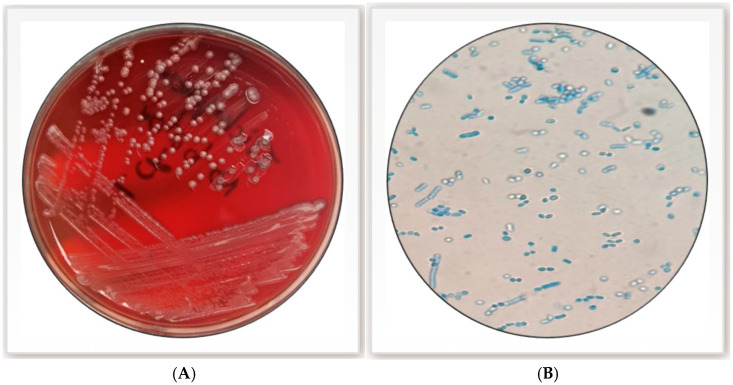
Macromorphological and micromorphological characteristics of a clinical *Trichosporon asahii* isolate case 5 (Mixed ICU, 81 years old, male): Deep ISO classified. (**A**) Growth on Blood Agar (Tryptic Soy Agar (TSA) with Sheep Blood) medium displaying cream-colored, moist colonies with a distinctive white farinose covering. (**B**) Lactophenol Cotton Blue stain demonstrating abundant arthroconidia alongside globose blastoconidia and true hyphae.

Microbiological, Phenotypic Characterization and Species Identification:

The isolates recovered from routine clinical samples initially displayed rapid growth on Blood Agar (TSA with Sheep Blood) media, developing characteristic cream-colored, moist, and wrinkled colonies with a distinctive white farinose covering over 48 to 72 h (Figure 1A). Microscopic examination of these colonies via lactophenol cotton blue staining revealed classic morphologic features, characterized by true hyphae, globose blastoconidia, and an abundance of rectangular arthroconidia formed by hyphal fragmentation (Figure 1B). Following this phenotypic screening, final species-level confirmation was performed using matrix-assisted laser desorption ionization–time of flight mass spectrometry (MALDI-TOF MS). Proteomic profile analysis confirmed that all nine clinical isolates (100%) were strictly identified as *Trichosporon asahii*, with high-confidence identification scores consistently ≥2.0, ensuring absolute taxonomic precision.

Regarding epidemiological indicators, the attack rate was 6.76%, and the overall mortality rate (from any cause among infected individuals) was 44%. Notably, the direct case fatality rate attributable to *T. asahii* was determined to be 20%, as confirmed by clinical mortality analysis and dedicated case review units, which verified that the fungal infection acted as a direct or underlying factor contributing to death in these specific cases. Analysis of shared risk factors revealed findings of high epidemiological consistency (Table 2).

The average length of hospital stay prior to the positive culture was 25 days (18–30 days), the average duration of mechanical ventilation prior to the event was 15 days (0–23.5 days), and the average duration of central venous catheter use prior to the event was 18 days (12–23.5 days).

Analysis of exposure frequency by antimicrobial class revealed that piperacillin-tazobactam was the most prevalent agent, used in 77.8% (n = 7) of cases, followed closely by vancomycin and meropenem, both at 66.6% (n = 6). Regarding prior antifungal therapy, 55.6% (n = 5) of patients received caspofungin before *T*. *asahii* isolation. Other broad-spectrum agents, such as trimethoprim-sulfamethoxazole and the ceftazidime/avibactam + aztreonam combination, were documented in 33.3% and 22.2% of patients, respectively. Only one patient (11.1%) had no record of prior exposure to broad-spectrum antibiotics during their stay.

The review of the 4795 supplies shared among the cases identified 35 common items, including intravenous catheters, bacterial filters, urinary drainage bags and feeding pump equipment, with no matches found in drug batches or health alerts from the Instituto Nacional de Vigilancia de Medicamentos y Alimentos—National Institute for Drug and Food Surveillance—(INVIMA) related to them.

All environmental cultures performed during the outbreak period (surfaces, biomedical equipment, infusion solutions, and air conditioning ducts) were negative. However, a thorough review of the ventilation system revealed two critical findings: (1) the absence of a current contract for the periodic cleaning of the air conditioning ducts, and (2) documented intermittent failures of the central refrigeration unit in the surgical ICU during the outbreak period, with temperature fluctuations not systematically recorded.

Following the outbreak declaration, the institutional protocol was immediately activated. This included the temporary closure of the surgical ICU and the establishment of a cohort of colonized or infected patients under strict contact precautions. Environmental interventions included terminal cleaning of cubicles with vaporized hydrogen peroxide (HPV) upon each patient’s discharge, inspection and maintenance of air conditioning filters, and weekly monitoring of microbial load on surfaces using luminometry. Additionally, hand hygiene monitoring was significantly intensified by strengthening the ward-monitoring program (vigías) in the intensive care units. The hospital epidemiology team increased on-site visits to provide education, feedback, and individualized field activities to improve compliance. Logistical barriers, such as a temporary shortage of disposable paper towels due to supplier issues, were resolved, and all glycerinated alcohol dispensers were inspected to ensure proper functionality and continuous availability at the point of care, and the use of ironed bed linens was ensured as a thermal control measure. Epidemiological control was strengthened with weekly active contact tracing through rectal swabs and urine cultures, complemented by a communication strategy that included weekly meetings with department heads, the infection control committee, and the assistant management, as well as direct coordination with the secretaria distrital—District Health Secretariat—Cali.

The outbreak was declared closed on 25 November 2024, one month after the last case (25 October 2024), with no new cases associated with the outbreak documented during the post-intervention surveillance period.

## 4. Discussion

This report describes what, to our knowledge, is the first documented hospital-acquired outbreak of *T. asahii* in Colombia. While nosocomial clusters due to this emerging pathogen remain rare globally, with only four events previously documented in the literature [7], its epidemiological and environmental characteristics provide valuable insights into the intrahospital ecology of *T. asahii* at a high-complexity institution.

The overall attack rate of 6.76% is consistent with outbreaks described in the literature, where the incidence varies between 3% and 10% in high-risk units [4]. The rigorous differentiation between the five cases of healthcare-associated infections (HAIs) and the four cases of colonization identified through active surveillance is a fundamental methodological element, since the colonization cases would not have been detected without a structured active surveillance program. This underscores the need to maintain such strategies in the context of *Trichosporon* spp. outbreaks.

The overall mortality rate of 44% among infected cases, with a direct case fatality rate of 20% attributable to *T. asahii*, is a finding that warrants detailed analysis. Although invasive trichosporonosis reported in the literature has mortality rates as high as 80% [3], our direct case fatality rate of 20% was strictly verified by clinical mortality analysis and dedicated case review units, confirming that the fungal infection acted as a direct or underlying factor contributing to death in these specific cases. While deceased patients presented severe underlying conditions (multi-organ failure, disseminated intravascular coagulation, and bacterial ventriculitis caused by multidrug-resistant organisms), this outcome demonstrates that *T. asahii* carries an independent lethal potential in critically ill, non-hematological patients, rather than being a mere marker of host comorbidity bystander status [8].

The presence of risk factors in most cases (prolonged hospital stay, use of invasive devices, and broad-spectrum antibiotics) is of great clinical and epidemiological relevance. Prolonged stay in the ICU facilitates the disruption of mucosal barriers, the selection of opportunistic flora, and repeated exposure to environmental sources. The use of broad-spectrum antibiotics promotes dysbiosis and eliminates the bacterial competition that normally limits yeast colonization [3,8]. These factors, consistently documented in our cohort, should be considered warning signs for the early implementation of an active mycological surveillance system in ICU patients.

When placing our experience in a global context, important differences in duration, setting, and magnitude emerge compared to historical cohorts. For instance, while large-scale clusters like the one reported in Brazil involved 70 patients over several years [6], or the outbreak in France spanned three years [9], our outbreak represented an acute, highly localized cluster restricted to a specific three-month period (August–October 2024). Furthermore, documented outbreaks in countries with high prevalence, such as India, have predominantly affected highly restricted settings like Neonatal ICUs with severe mortality (e.g., 6 deaths out of 8 patients, or 3 deaths out of 3 patients) [10]. In contrast, our cluster occurred entirely within an adult Surgical ICU, expanding the understanding of *T. asahii* transmission dynamics in broader critical care populations.

Finally, the challenges encountered during our environmental investigation mirror international precedents. In some historical outbreaks, specific medical devices were identified as vehicles [11], but in major clusters in France [9] and Brazil [6], the definitive source remained completely unknown despite extensive testing.

The hypothesis of an environmental reservoir linked to the ventilation and air conditioning system emerges as a plausible epidemiological explanation for this outbreak, although it lacks direct microbiological or molecular confirmation. The negative environmental cultures (including those taken from ventilation ducts) could be explained by a low fungal inoculum, the intermittent nature of the dispersal phenomenon (dependent on equipment failures), and the technical limitations of duct sampling [12].

*Trichosporon* spp. can survive on dry surfaces and proliferate in fluctuating temperatures and residual humidity in ventilation ducts; when equipment operation is restored, airflow can act as a vector for the dissemination of fungal particles [13]. Without definitive genomic sequencing to confirm clonal relatedness between patient and environmental strains, this association remains an unverified microbiological link. However, the absence of a duct cleaning contract, the documented failure of the refrigeration unit in the Surgical ICU—the unit with the highest concentration of cases—and the immediate cessation of cross-transmission after ventilation system interventions strongly reinforce this epidemiological hypothesis.

From a treatment perspective, it is worth noting that *T*. *asahii* exhibits intrinsic resistance to echinocandins and variable sensitivity to azoles. Due to limited clinical evidence, the treatment of *Trichosporon* spp. infections is based primarily on experimental studies and case series, where voriconazole regimens have shown superior efficacy to amphotericin B, with posaconazole and isavuconazole emerging as viable alternatives. The initial use of combination therapies (azoles with polyenes or echinocandins) is not recommended, reserving them only for rescue cases [14]. Systematic fungigram testing of all outbreak isolates is essential to guide individualized therapeutic decisions and detect potential emerging resistance patterns.

Regarding control measures, the combination of unit closure, HPV decontamination, strict cohorting, and active surveillance proved effective in containing transmission within four weeks of implementation. HPV has demonstrated superiority over conventional disinfectants in eliminating fungi resistant to adverse environmental conditions, including *Trichosporon* spp. [15].

Our study has some limitations that warrant consideration. First, it is important to acknowledge that the isolation of *T. asahii*. from a sterile site does not invariably indicate active clinical infection or true colonization. Due to the challenges inherent in medical or laboratory procedures, the risk of sample contamination during collection or processing cannot be entirely ruled out. Consequently, distinguishing between true opportunistic pathogen involvement and transient pre-analytical contamination remains a critical diagnostic challenge in epidemiological investigations of this nature. Second, a major limitation is the lack of molecular characterization (such as whole-genome sequencing or microsatellite typing) to confirm clonal relatedness between patients or with potential environmental sources [6,9]. Consequently, a definitive environmental source could not be verified microbiologically, and our findings regarding the air conditioning system must be interpreted strictly as an epidemiological hypothesis based on temporal and spatial association.

## 5. Conclusions

This nosocomial outbreak of *T*. *asahii* in a high-complexity hospital in Cali, Colombia, offers the following lessons:

Environmental engineering as a cornerstone of prevention: Rigorous maintenance of air conditioning systems in ICUs should not be considered a secondary technical task, but rather a strategic priority for infection control. It is imperative to move towards management models with auditable schedules and formal contracts to mitigate the risk of fungal reservoirs.

Thermal and humidity biosurveillance: Continuous monitoring of in-hospital climate variables is a highly cost-effective intervention. This measure allows for the early detection of microclimates that favor the proliferation of emerging pathogens before they become clinically apparent.

Active surveillance in outbreak control: While there is no current evidence supporting the use of antifungal prophylaxis to halt *T. asahii*. transmission—and thus it was not indicated or administered in this outbreak—active screening proved to be essential for epidemiological containment. The primary value of this strategy lay in identifying asymptomatic colonized patients, which enabled the characterization of their individual risk factors and immediate surroundings. This data was critical for implementing targeted infection prevention strategies, effectively stopping clinical cross-transmission.

Beyond Mortality: A marker of vulnerability, although a direct case fatality rate of 0% was achieved, the detection of *T*. *asahii* should be interpreted as a sentinel of extreme host fragility. Its presence should prompt a critical re-evaluation of the use of broad-spectrum antibiotics, nutritional support, and the continued use of invasive devices.

The Multidisciplinary Response Model: The success in containing this outbreak underscores that collaborative clinical governance—integrating hospital epidemiology, infectious diseases, nursing, microbiology, and engineering—is the only effective way to address the challenges of modern hospital ecology.

## Figures and Tables

**Table 1:** Epidemiological profile of patients with *Trichosporon asahii* isolation during an outbreak in a high-complexity university hospital, Cali, Colombia (16 August–25 October 2024).

Case	Ward/Unit	Age/Sex	Type/Date of Sample Collection	Time of Isolation (days)	Classification	Underlying Comorbidities	Co-Infections	Risk Factors	Outcomes	Length of Stay (days)
1	ID-ICU	58/M	Blood culture/24/08/14	27	HAI—Fungemia	- Bipolar affective disorder. - Nursing home resident.	Multilobar pneumonia.	CVC, MV, BSA, prolonged LOS, AKI, malnutrition, pleural and abdominal drains	Deceased	28
2	S-ICU	32/M	Peripancreatic collection, peritoneal membranes, and peritoneal fluid/24/08/21	18	HAI—Organ/space SSI	None	Intra-abdominal infection.	CVC, MV, BSA, prolonged LOS, AKI, multiple surgeries, TPN, thoracostomy	Survived	62
3	S-ICU	52/F	CSF/24/08/26/	33	HAI—Organ/space SSI	- Hypertension - Type 2 diabetes mellitus - Acute kidney injury requiring hemodialysis	Intractable ventriculitis with hydrocephalus; *P. aeruginosa* isolated from CSF culture; Herpes simplex and Cytomegalovirus detected on meningitis/encephalitis panel	CVC, MV, BSA, prolonged LOS, multiple surgeries, ventriculostomy, gastrostomy, tracheostomy	Deceased	71
4	Trasplant ward	68/F	Rectal swab/24/08/24	25	Colonization	-Type 2 diabetes mellitus - Moderately differentiated and infiltrating adenocarcinoma of the ampulla of Vater	Rectal screening with simultaneous isolation of *C. glabrata*; polymicrobial peritonitis: *A. hydrophila*, *E. casseliflavus*/*gallinarum*	CVC, prolonged LOS, underlying malignancy, abdominal surgery, abdominal drain	Survived	37
5	S-ICU	37/M	Abdominal collection/24/09/05	21	HAI—Organ/space SSI	Psychoactive substance use	Bacteremia due to *B. cepacia*, *A. baumannii*, and *K. pneumoniae*	CVC, MV, BSA, prolonged LOS, multiple surgeries. - Gunshot wound with multiple perforations of the small intestine and sigmoid colon, neck and extremity wounds, hemoperitoneum, and pelvic hematoma	Survived	47
6	C-ICU	52/M	Urine culture/24/09/20	25	Colonization	- B-cell lymphoma with orbital involvement. - Cervical spinal stenosis due to old odontoid fracture with C1–C2 compressive myelopathy. - Hypothyroidism	Pneumonia due to *A. calcoaceticus/baumannii*, *K. pneumoniae*, and *Proteus* spp. detected on pneumonia panel	Prolonged LOS, underlying malignancy, malnutrition, steroid use (dexamethasone)	Deceased	88
7	Surgical ward	60/F	Urine culture/24/09/20	41	Colonization	- Stage IVA endometrial cancer with ureteral and bladder involvement. - AKI requiring hemodialysis.	Secondary bacteremia due to complicated urinary tract infection by *K. pneumoniae* and *P. aeruginosa*	CVC and PICC, prolonged LOS, BSA, malnutrition, underlying malignancy, pelvic surgery, nephrostomies, colostomy.	Deceased	102
8	Internal Medicine ward	42/M	Urine culture/24/09/29	19	Colonization	Pemphigus vulgaris with 70% TBSA involvement.	Urinary tract infection by *P. aeruginosa* 12 days prior	PICC, BSA, prolonged LOS, steroid and rituximab use	Survived	36
9	Mixed ICU	80/M	Tissue/secretion/24/10/24	15	HAI—Deep incisional SSI	Obesity	Simultaneous isolation of *P. aeruginosa* in the same sample	CVC, MV, BSA, prolonged LOS, AKI requiring hemodialysis, multiple escharectomies, 30% TBSA burn.	Survived	124

AKI: Acute kidney injury; BSA: Broad-spectrum antibiotics; C-ICU: Cardiovascular ICU; CKD: Chronic kidney disease; CSF: Cerebrospinal fluid; CT: Chest tube; CVC: Central venous catheter; F: Female; ID-ICU: Infectious diseases ICU; LOS: Length of stay; M: Male; MV: Mechanical ventilation; S-ICU: Surgical ICU; TBSA: Total body surface area; TPN: Total parenteral nutrition.

**Table 2:** Identified Risk Factors Among 9 Cases in a *Trichosporon asahii* Outbreak, August–October 2024.

Risk Factor	Cases (n)	Percentage (%)	Category
Male sex	6/9	66.7%	Demographic
LOS > 7 days	9/9	100%	Structural
Endotracheal intubation	6/9	66.7%	Procedure
CVC	9/9	100%	Procedure
Prior broad-spectrum antibiotics	8/9	88.9%	Pharmacological
Immunosuppression-associated diagnoses *	4/9	44.4%	Host
Medical-surgical procedures (<90 days)	6/9	66.7%	Procedure
Transfer from another facility	5/9	55.6%	Epidemiological

CVC: central venous catheter; LOS: length of stay. * Immunosuppressive conditions: Solid tumors (infiltrating adenocarcinoma of the ampulla of Vater, metastatic stage IVA endometrial cancer); hematological malignancies (orbital tumor consistent with diffuse large B-cell lymphoma); bullous impetigo.

## Data Availability

The data presented in this study are available upon request from the corresponding author. The data are not publicly available due to institutional privacy and confidentiality policies regarding medical records.

## Abbreviations

The following abbreviations are used in this manuscript:

ACIN	Asociación Colombiana de Infectología
AKI	Acute kidney injury
BSA	Broad-spectrum antibiotics
C-ICU	Cardiovascular intensive care unit
CDC/NHSN	Centers for Disease Control and Prevention—National Healthcare Safety Network
CKD	Chronic kidney disease
CSF	Cerebrospinal fluid
CT	Chest tube
CVC	Central venous catheter
F	Female
HAI	Healthcare-associated infections
HPV	Vaporized hydrogen peroxide
ID-ICU	Infectious diseases intensive care unit
INS	Instituto Nacional de Salud
INVIMA	Instituto Nacional de Vigilancia de Medicamentos y Alimentos
LOS	Length of stay
M	Male
MV	Mechanical ventilation
S-ICU	Surgical intensive care unit
TBSA	Total body surface area
TPN	Total parenteral nutrition
